# Vortex Whistle and Smart Phone Application for Peak Flow Recordings in Asthmatic Children: A Feasibility Study

**DOI:** 10.1089/tmj.2018.0270

**Published:** 2019-11-06

**Authors:** Ingvild Bruun Mikalsen, Damoun Nassehi, Knut Øymar

**Affiliations:** ^1^ Department of Pediatrics, Stavanger University Hospital, Stavanger, Norway.; ^2^ Department of Clinical Science, University of Bergen, Bergen, Norway.; ^3^ digiDoc Technologies, Egersund, Norway.

**Keywords:** *asthma*, *child*, *peak expiratory flow*, *telemonitoring*, *smart phone application*, *telemedicine*, *e-health*

## Abstract

**Introduction:** Variable airflow obstruction that can be confirmed by diurnal variability of peak expiratory flow (PEF) >13% is an important characteristic of asthma. Home monitoring of PEF may be helpful to diagnose and monitor asthma. In this feasibility study, we aimed to study if asthmatic children can measure PEF at home twice daily during a 4-week period using a device designed as a “whistle” and a smart phone software application.

***Materials and Methods:** Twice daily during 4 weeks, children aged 5–12 years with current asthma rated their asthma condition electronically on the smart phone application* Blowfish *before inhaling deeply then exhaling into the device to produce a high-pitched sound recorded by the application. Through mathematical algorithms, the sound was transferred to PEF, which was uploaded to a server. At inclusion, the Pediatric Asthma Quality of Life Questionnaire and the Childhood Asthma Control Test were answered. At the end, the parents graded the device and application.*

**Results:** One child did not manage to upload PEF. For the remaining 21 children, the median (quartiles) days with at least one measurement during the period were 27 (21–29.5), and on median 18 (9–24) days PEF was recorded twice daily. The median parental score (potential score 0–20) of the application was 18 (15–20).

**Discussion/Conclusion:** The study shows promising results for home monitoring of PEF by an electronic device with automatic teletransmission. The high rate of successful recordings and parental satisfaction suggests that the clinical utility of the solution should be further studied.

## Introduction

Asthma is characterized by airway inflammation, a history of airway symptoms such as wheeze, cough, and shortness of breath, and variable airflow obstruction.^1,2^ Airflow variability may be confirmed by >12% increase in forced expiratory flow in 1 second (FEV_1_) after inhaling a bronchodilator or after 4 weeks of anti-inflammatory treatment.^1^ However, these features may not always be present or easy to assess in children with asthma, and airflow variability may also be confirmed by diurnal variability of peak expiratory flow (PEF) >13%.^1^ Compared to adults, there is less evidence to support the routine use of PEF recording in the diagnosis and monitoring of asthma in children.^2^ In addition, both multiple and daily PEF recordings are needed to obtain reliable estimates of air flow variability, and monitoring airflow variability by PEF therefore requires good compliance.^2^ Compliance and accuracy are challenging in home spirometry,^3^ but may be improved using electronic peak flow meters.^4^

Smart phone applications with features such as diaries, reminders for medications, places to document triggers, and PEF monitoring are associated with increased knowledge and ability to self-judge the patient's asthma severity.^5^ Mobile phones and web based applications offer new possibilities for guided self-management,^6^ and a Cochrane review from 2011 concluded that telehealthcare has the potential to reduce the risk for hospital admissions for asthma.^7^

A novel three-dimensional printed vortex whistle using a smartphone's built-in microphone as a spirometer has been designed and evaluated as a useful portable alternative to clinical spirometry for managing moderate airway obstruction.^8^ The aim of this observational study was to assess the feasibility for children with asthma to use the vortex whistle and smart phone application to measure PEF twice daily during a 4-week period.

## Methods

### Device and Application

**Fig. 1.**
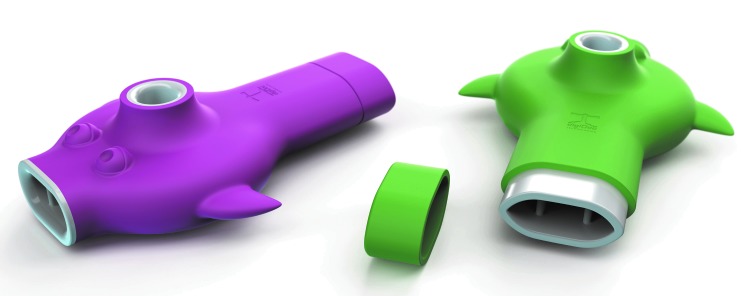
The device is a plastic vortex whistle consisting of an inlet, a circular main chamber, and a vertical outlet.

The device consists of a combination of hardware and software. The hardware is a plastic whistle consisting of an inlet, a circular main chamber, and a vertical outlet (Fig. 1). The software is an application named *Blowfish* running on a commercial smartphone (*Apple's iPhone*). The hardware device is a “vortex whistle,” which emits a frequency that is directly determined by the air flow rate passing through the whistle. Air is blown into the inlet, flows to the main chamber, where the air is compressed as it whirls around in a vortex and eventually exits through the vertical outlet on the top of the main chamber. As air enters the outlet, the whistle produces a sound. The pitch varies directly with the inlet air flow rate. The frequency can then be mapped to the inlet airflow rate, allowing for PEF measurement. Sound capture is performed by the smartphone microphone, and several software algorithms are applied to first estimate the frequency and harmonic following, then linear multivariate ridge regression is applied to find the flow rate, and eventually PEF.^8^ The application collects and uploads the data automatically to a secure cloud server managed by digiDoc Technologies (Egersund, Norway). The devices' digital connectivity is advantageous compared to a regular PEF meter, as it enables data aggregation, analysis, and improved treatment support.

### Participants

We invited children aged 5–12 years with current asthma and at least one parent with an iPhone to participate in the study. Current asthma was defined as a positive answer to the following questions: (1) Wheezing or whistling in the chest or chest tightness during the preceding 12 months and (2) Use of asthma medication (controller and/or rescue medication) during the preceding 12 months. The diagnosis was verified by one of the authors (IBM or KØ) at a regular visit to the outpatient clinic at Stavanger University Hospital. Exclusion criteria were current neurological, temporomandibular, cognitive, or other problems, which could interfere with lung function testing.

### Study Design

**Fig. 2.**
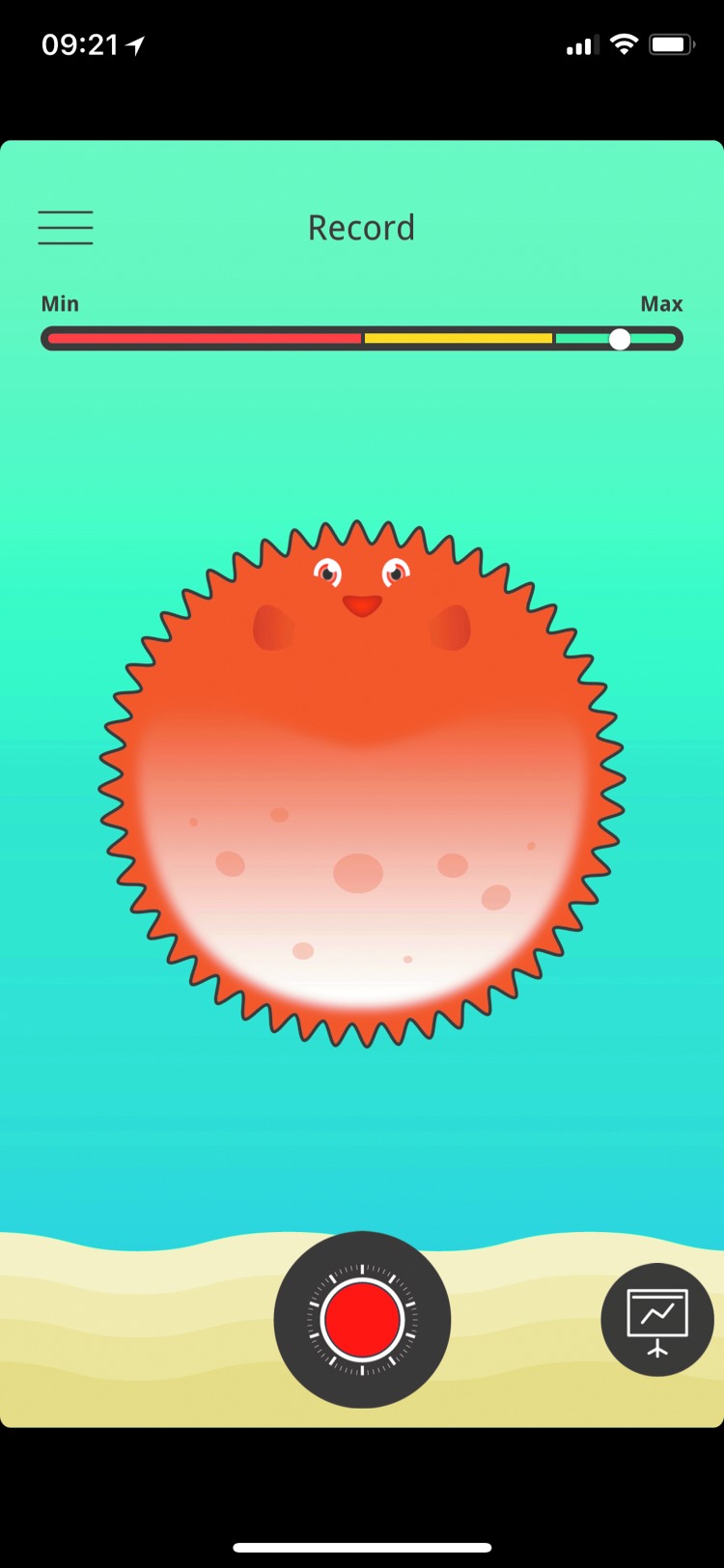
The figure shows the wheel that the participants had to turn on the application to rate their asthma condition from minimum to maximum.

**Fig. 3.**
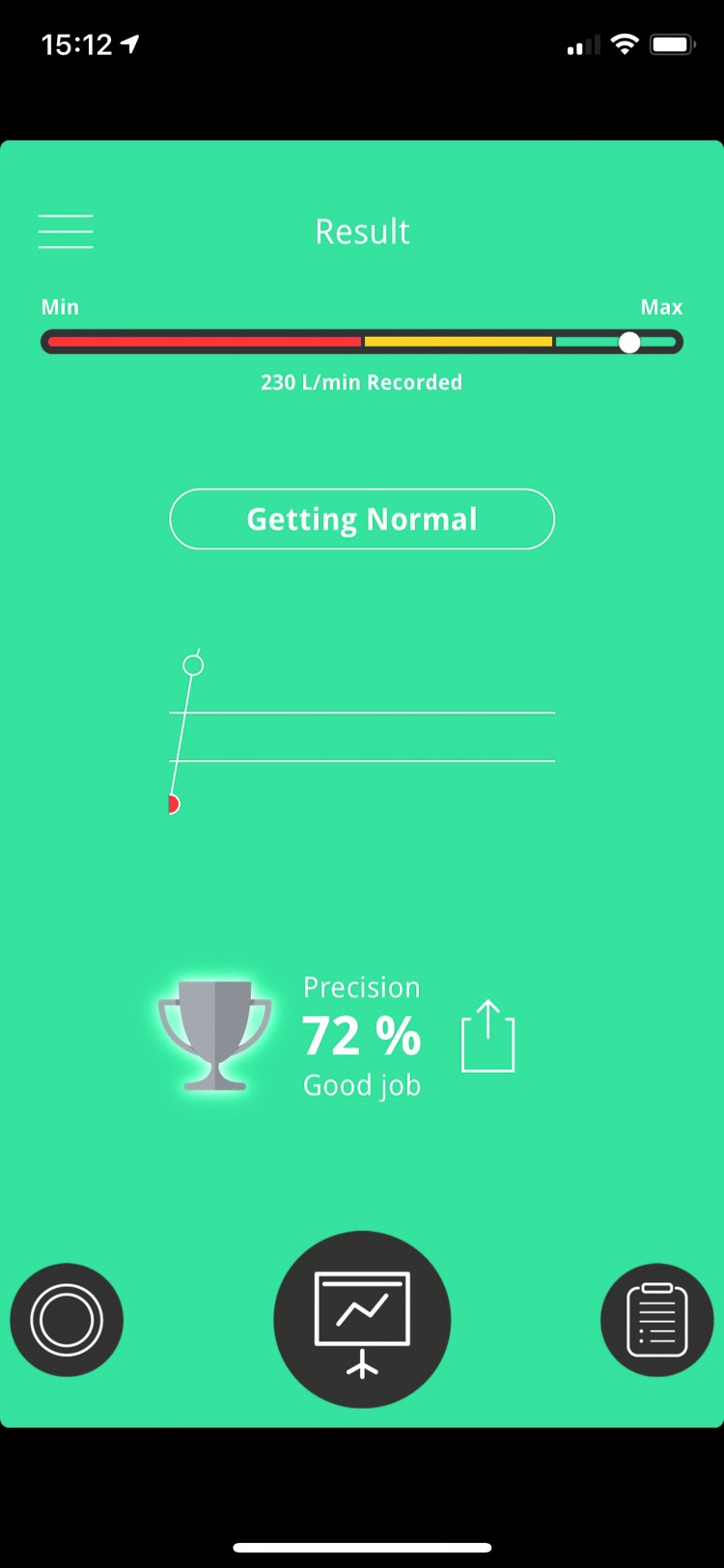
The figure shows an example of how the participants got visual feedback (rated as percentage) on how they performed compared to their own prior prediction and compared to reference values (indicated by red, yellow, or green background). In the present example, the peak expiratory flow was 230 L/min, which was 72% of their prior prediction.

All children received a vortex whistle as shown in Figure 1, and the asthma whistle application *Blowfish* was downloaded to the parents' or children's iPhone. Date of birth, sex, weight, and height were entered into the application. The participants were instructed to take a deep inhalation and then blow as strongly as possible into the asthma whistle device to produce the most high-pitched sound as possible. The children should blow at least twice daily; morning and evening for 4 weeks. Before blowing, the participants had to rate their asthma condition from minimum to maximum by turning a wheel on the application (Fig. 2). After blowing, the participants got direct visual feedback (rated as percentage) on how they performed compared to their own prior prediction and also compared to reference values (Fig. 3).

The children and their parents answered the Pediatric Asthma Quality of Life Questionnaire (PAQLQ) at study start.^9^ In addition, the parents and children were asked to fill in the Childhood Asthma Control Test (C-ACT).^10^ The C-ACT contains seven selected items; three items were answered by the children and four by the parents. The children completed the questionnaire at the end of each week a total of four times (C-ACT 1–4), while the parental questionnaire collects symptoms during the last 4 weeks and was therefore only answered at the end of the 4-week study period. All the questionnaires were paper based and collected at the end of the study period. The potential range in score was 0–12 and 0–15 for the children and parental questionnaire, respectively. At the end of the study period, the children questionnaire from week 4 (C-ACT 4) and the parental questionnaire were summarized to obtain a total score for the C-ACT, with a potential range in score from 0 to 27. A total C-ACT score ≥20 indicates a well-controlled asthma.^10^ At the end of the study period, the parents also graded the user friendliness and functionality of the application, their assessment of the whistle, and their overall assessment of the whistle and application. Each question had a potential range from 0 to 5, summarized to total score with a potential range from 0 to 20.

The children and parents were informed that the *Asthma Whistle* is under development and that no change in treatment should be done during the study period without consulting a doctor.

The results are presented as median and interquartiles. Analyses were performed using SPSS version 24.0 (IBM Corp., Armonk, NY).

The study was approved by the Regional Committee on Medical Research Ethics. Signed statements of informed consent were obtained from the participants' parents.

## Results

Twenty-two children (*n* = 22) aged 5–12 years with physician-diagnosed asthma were included. Baseline characteristics of all children are given in Table 1. The overall median (quartiles) PAQLQ score was 6.4 (6.1–6.7) (Table 1). The children had median (quartiles) C-ACT total score after 4 weeks of 24 points (15.5–27) and were assessed to have a well-controlled asthma.

**Table 1.** Baseline Characteristics of the 22 Children Included in the Study

VARIABLE	MEDIAN (QUARTILES)^a^	POTENTIAL SCORE	NUMBER OF RESPONDENTS WITH VALID ANSWERS
Children included (boys/girls)			22 (16/6)
Age years; mean (SD)	10.2 (2.2)		22
C-ACT-1	10 (10–11)	0–12	19
C-ACT-2	11 (10–12)	0–12	19
C-ACT-3	11 (9–12)	0–12	17
C-ACT-4	11 (7–12)	0–12	17
C-ACT-parents	13 (9–15)	0–15	19
C-ACT-total	24 (15.5–27)	0–27	17
PAQLQ			
Overall quality of life	6.4 (6.1–6.7)	1–7	15
Symptoms	6.4 (5.4–6.5)	1–7	19
Activities	6.2 (5.8–6.6)	1–7	15
Emotions	6.6 (6.0–6.9)	1–7	19

^a^ Apart from age, the results are presented as median (quartiles).

1 point, severe impairment; 7 points, no impairment; PAQLQ, Pediatric Asthma Quality of Life Questionnaire.

C-ACT1–4: childhood asthma control test at week 1–4. C-ACT-total contains C-ACT-4 summarized with C-ACT-parents. C-ACT total score ≥20 indicates a well-controlled asthma.

One child had no successful uploaded recordings of PEF. For the remaining 21 children, the median (quartiles) days with at least one measurement during the 4-week period were 27 (21–29.5). Median days with recordings both morning and evening were 18 (9–24), with 22 (15.5–26) days with recordings in the morning and 24 (17.5–28) days with recordings in the evening (Table 2).

**Table 2.** Number of Successful Peak Expiratory Flow Recordings and Parental Assessment of the Application, During a 4-Week Period in 21 Children Aged 5–12 Years with Physician-Diagnosed Asthma

VARIABLE	MEDIAN (QUARTILES)	NUMBER OF RESPONDENTS WITH VALID ANSWERS
Days with at least one measurement, morning or evening	27 (21–29.5)	21
Days of measurements both morning and evening	18 (9–24)	21
Days of measurements morning	22 (15.5–26)	21
Days of measurements evening	24 (17.5–28)	21
Parental 1 (application user friendliness)	5 (4–5)	15
Parental 2 (application functionality)	4 (3–5)	15
Parental 3 (assessment of whistle)	5 (4–5)	15
Parental 4 (overall assessment of whistle and application)	4 (4–5)	14
Total parental score of application	18 (15–20)	14

Parental 1–4: Parental assessment of the application, potential score was 0–5 for Parental 1–4 and 0–20 for Total parental score of the application.

The median (quartiles) parental score of the application was 18 (15–20) (Table 2).

## Discussion

As previous published, this electronic device has shown promising results as a PEF meter.^8^ According to guidelines from Global initiative for asthma, an excessive variability in twice-daily PEF >13% during 2 weeks may be helpful in confirming the asthma diagnosis.^1^ In addition, short-term PEF monitoring may be used to assess response to treatment, evaluate asthma triggers, and establish a baseline for action plans. Excessive variation in PEF suggests suboptimal asthma control.^1^ However, twice daily PEF measurement is difficult to achieve and requires a high degree of compliance, but may be improved using electronic PEF meters combined with daily feedback from medical staff during telemedicine.^4^ Such electronic solutions may thereby increase the impact of PEF in confirming the asthma diagnosis and monitoring of asthma in children.

In this feasibility study, 21 of 22 children with well-controlled asthma managed to record PEF by the use of an asthma whistle device and a smart phone application, and the data were successfully uploaded to a secure server. During the 4-week long study period, most children had at least one daily successful recording, but not all children managed to blow twice daily. The total parental score of the application was high, indicating that most parents were very satisfied with the device and the application. The high rate of children managing the technological challenges and the high rate of parental satisfaction is in line with a study, including asthmatic adult patients performing internet based asthma telemonitoring, which found no difference between unsupervised and supervised home spirometry and successful implementation also in patients with no computer experience.^11^

To our knowledge, this is the first electronic PEF meter with automatic teletransmission and with possibilities for telemonitoring. Electronic PEF meters with local data storage are already available, and these electronic devices show considerable less variation and more correct entries than those reported by conventional written peak flow diaries.^4,12,13^ However, the impact of telemedicine on monitoring asthma in children is not properly studied due to various and still increasing number of new devices and solutions. In a French study, telemonitoring of FEV_1_ with daily medical feedback in children with severe asthma did not reduce the number of severe exacerbations.^14^ In contrast, improved asthma control and FEV_1_ were found in a group of asthmatic adults offered daily short message service (SMS) reminders of asthma action plans and PEF monitoring compared to those not receiving daily SMS reminders.^15^ However, no difference in asthma control test (ACT) was found in a similar study, including one group of adults reporting PEF and ACT by SMS and thereafter receiving a response on the reported values, compared to a group not reporting results by SMS or receiving such feedback.^16^ A systematic review concluded that health care through cell phones using standard care with reminders, disease monitoring and management and education through cell phone voice and SMS, can help improve health outcomes and care processes for various clinical areas.^6^

A Cochrane review from 2011 concluded that telehealthcare may reduce admissions to hospitals, but do not appear to be more able to improve quality of life than routine models of care nor reduce the number of visits to the emergency department.^7^ In this Cochrane review, telehealthcare was defined as electronic transfer of information, for instance, by telephone, internet, videoconference, or any other networked mobile device, which may be different from the solution described in the present study. Overall, it is difficult to compare the results from different electronic devices, but increasing number of new solutions requires future studies that can further assess the impact of these new electronic PEF meters in diagnosing and monitoring asthma in children.

The present device and software also offer the possibilities of direct feedback to the participants, both compared to their prior prediction and compared to reference values. These features were not tested in the present feasibility study. Feedback involving direct detection of airflow obstruction and symptoms is effective to identify asthma triggers and may decrease underperception of respiratory symptoms and increase adherence to asthma medications.^6,17–19^ In addition, in this particular application, asthma action plans can be downloaded making the physicians able to offer daily evaluation and adjustment of treatment with a possible clinical benefit as shown in another study.^20^ In one study, children given a written printed asthma plan and instructed to self-manage adjustment according to PEF values and symptoms, knowledge of PEF did not enhance self-management even during exacerbations^21^; this challenge could be overcome by direct daily/weekly feedback by their physician as offered in the present device and application.

As validation of the device as a PEF meter was not the aim of the study, the importance of correct blowing technique was not stressed for the participants and the children were not instructed to blow more than once morning and evening. Instructing the children to blow more correctly could have improved the overall quality of the measurements and increased the number of successful recordings. It is also important to underline that the children in the study had well-controlled asthma and therefore probably high adherence, and it is therefore likely that the number of successful recordings would be lower if the study was performed in a group of children with less-controlled asthma. As this was a feasibility study, only 22 children were invited to participate, limiting the overall importance of the results.

In conclusion, this feasibility study shows promising results for PEF measurements using a newly designed electronic asthma whistle device and smart phone application with a high rate of successful recordings and high parental score. Taken together with the positive results from other telemedicine studies, future studies should determine if electronic PEF meters offering automatically upload to medical staff with possibilities of direct feedback might be helpful in diagnosing and monitoring asthma in children.
